# Effect of Replacing Sorghum Stubble with *Tillandsia recurvata* (L.) on Liveweight Change, Blood Metabolites, and Hematic Biometry of Goats

**DOI:** 10.3390/biology11040517

**Published:** 2022-03-28

**Authors:** Héctor G. Gámez-Vázquez, César A. Rosales-Nieto, Jorge Urrutia-Morales, Miguel Mellado, César A. Meza-Herrera, Juan M. Vázquez-García, Luisa E. S. Hernández-Arteaga, Luis O. Negrete-Sánchez, Catarina Loredo-Osti, Marco A. Rivas-Jacobo, Sergio Beltrán-López

**Affiliations:** 1 Instituto Nacional de Investigaciones Forestales, Agrícolas y Pecuarias, Campo Experimental San Luis, San Luis Potosí 78431, Mexico; gamez.hector@inifap.gob.mx (H.G.G.-V.); urrutia.jorge@inifap.gob.mx (J.U.-M.); 2 Facultad de Agronomía y Veterinaria, Universidad Autónoma de San Luis Potosí, San Luis Potosí 78321, Mexico; cesar.rosales@uaslp.mx (C.A.R.-N.); manuel.vazquez@uaslp.mx (J.M.V.-G.); socorro.hernandez@uaslp.mx (L.E.S.H.-A.); luis.negrete@uaslp.mx (L.O.N.-S.); catarina.loredo@uaslp.mx (C.L.-O.); marco.rivas@uaslp.mx (M.A.R.-J.); 3 Departamento de Nutrición Animal, Universidad Autónoma Agraria Antonio Narro, Saltillo 25315, Mexico; melladomiguel07@gmail.com; 4 Unidad Regional Universitaria de Zonas Áridas, Universidad Autónoma Chapingo, Texcoco 35230, Mexico; cmeza2020@hotmail.com; 5 Instituto de Investigaciones de Zonas Desérticas, Universidad Autónoma de San Luis Potosí, San Luis Potosí 78377, Mexico

**Keywords:** extensive system, feed intake, plasma total proteins, plasma glucose, mycotoxins

## Abstract

**Simple Summary:**

*Tillandsia recurvata* is an epiphyte that contains adequate protein and energy levels regardless of the season and can be used as forage for small ruminants. We evaluated the effects of substituting sorghum stubble for *T. recurvata* in adult goats for 89 d, including an adaptation period of 14 d, at three different levels: 0% (control), 30%, and 60%. At the last phase of the trial, control goats gained more weight than goats consuming *T. recurvata*. Overall, the metabolic status was similar among treatments, whereas some variables tested of the complete blood count differed among treatments. Interestingly, the inclusion of *T. recurvata* in goats’ diets at 30% gained or at 60% decreased 1% live weight; without signs of health disturbance. Using up to 60% *T. recurvata* in the diet did not negatively affect animal health; however, under the conditions of this study, supplemental protein would be required when *T. recurvata* completely replaced sorghum stubble. The use of *T. recurvata* as a supplement for goats would improve the nutrition of grazing goats in the dry season compared to the current diet obtained from arid rangelands and would alleviate host trees invaded by this aggressive epiphyte.

**Abstract:**

*Tillandsia recurvata* is an epiphyte that grows on the canopy of many trees in tropical and subtropical areas of America. The objective of this study was to evaluate the effect of partial or complete substitution of sorghum stubble with *T. recurvata* on liveweight change, metabolic profile, and complete blood count of goats fed increasing levels (0, 30, and 60%, dry matter basis) of *T. recurvata*. Thirty non-pregnant three-year-old, non-lactating, healthy mixed-breed goats, ten animals per treatment (T0, T30, and T60), were adapted to diets and facilities for 14 days (d-14). Blood samples were collected at d-15, 28, and 56. At the last phase of the trial (from days 67 on), control goats tended to gain more (*p* = 0.09) weight than their counterparts consuming *T. recurvata.* Plasma protein, glucose, triglycerides, calcium, and phosphorus concentrations did not differ among dietary treatments (*p* > 0.05). Dietary treatment influenced red blood cells (higher for T60; *p* < 0.01), white blood cells (higher for T30; *p* < 0.05), mean corpuscular volume (higher for T0; *p* < 0.001), and mean corpuscular hemoglobin concentration (higher for T0; *p* < 0.01), although not the rest of the blood variables (*p* > 0.05). The hematocrit percentage tended to be higher (*p* = 0.06) in T30 than T0 and T60. It was concluded that replacing sorghum stubble with *T. recurvata* did not modify the metabolic status and maintained live weight of goats. Nevertheless, the use of *T. recurvata* as feed for goats would improve the nutrition of these animals in the dry season compared to the current diet obtained from an arid rangeland, reducing production costs, and would alleviate the damage caused by this aggressive epiphyte to host trees.

## 1. Introduction

Small ruminants are an integral and important part of farming systems in developing countries with extensive arid ecosystems and play a transcendental role in poverty alleviation and increased food security in rural communities [1]. Rangelands of these regions are often overgrazed, with poor-quality soils and low and highly variable annual rainfall [2]. Accordingly, forage supply varies from year to year, and crop production is unreliable, and this scenario is becoming more pronounced with climate change. Livestock raised under these conditions generally does not receive feed supplements due to the high prices of high-quality pasture and concentrate for smallholders [3].

There are more than 8.8 million goats in Mexico, with a predominance of mixed-breed animals, primarily raised in xeric ecosystems [4]. It has been demonstrated that the nutrient content of forages in rangelands in the dry season does not meet the nutritional requirements of gestating and lactating goats [5,6]. Nevertheless, goats thrive in desert rangelands due to their particular anatomic characteristics, browsing ability, and wide diet selection [7]. Additionally, goats use forages with a wide array of secondary compounds without experiencing toxic effects, which help them cope with the available vegetation′s nutritional deficiency [6,8,9]. Thus, goats adapt well to terrains with scarce forage resources and still are capable of presenting an acceptable reproduction rate and producing moderate levels of milk and meat [6,10,11,12]. Rangelands in northern Mexico compose around 126 plant species, including annual plants, forbs, grasses, perennial herbs, shrubs, and trees [5,6,7]. Important species found in this landscape are *Acacia berlandieri*, *Acacia greggii*, *Aristida arozonica*, *Bouteloua dactyloides*, *Bouteloua gracilis*, *Croton dioicus*, *Muhlembergia repens*, *Prosopis glandulosa*, *Sida abutifolia*, *Solanum elaeagnifolium*, *Sphaeralcea angustifolia*, and *Vachellia farnesiana* [5,6,7].

In these marginal regions where forage is scarce in the dry season, some alternative supplements have been proposed, such as spineless cactus to cope with the nutritional deficiency of the available vegetation [13,14,15]. Goats in xeric ecosystems consume *Tillandsia usneoides* in the dry season [6]. However, it is unknown if consumption of this epiphyte may be harmful to goats if ingested in greater quantity than previously reported [6]. García-Monjaras et al. [6] reported that goats with different milk yield potential included *T. unsenoides* in their diet during winter (5%) and spring (0.2%). *Tillandsia* spp. are an epiphyte that belongs to the family of the Bromeliaceae and are widely distributed across the American continent [16], including semi-arid shrublands [17]. *Tillandsia* spp. attach on to the surface of the trunks and branches of various host trees [18,19,20] and, depending on the rate of invasion, may cause abscission of leaves, reducing leaf growth [21,22]. Thus, goat production would benefit from consuming this epiphyte with the concurrent recovery of the infested trees [6,12,19].

It has been demonstrated that *Tillandsia recurvata* contains adequate protein, energy, and minerals for small ruminants, regardless of the location and season, making it suitable for feeding small ruminants in arid and semi-arid regions [23]. The abundance of *T. recurvata* in the Chihuahuan Desert, Mexico, has been reported to be as high as 73% of the observed individuals [22]. Females with adequate energy and protein levels when mating are more likely to get pregnant and increase prolificacy [15,24,25,26]. Thus, under a scarcity of forage, *T. recurvata* could be used as a supplemental feed for goats to alleviate malnutrition. However, it is unknown whether a high intake of this epiphyte has a detrimental effect on the metabolic profile, liveweight change, and, eventually, reproductive performance of goats in arid zones. Therefore, the objective of this study was to evaluate the effect of partial or complete substitution of sorghum stubble with *T. recurvata* on live animal performance, metabolic profile, and blood components of mixed-breed goats managed under marginal production systems on semi-arid rangeland.

## 2. Materials and Methods

### 2.1. Study Site

The study was conducted at the National Institute of Agricultural, Livestock and Forestry Research (INIFAP), located in Soledad de Graciano Sanchez, San Luis Potosi, Mexico (22°14′03″ N, 100°53′11″ O and 1835 m above sea level). The climate in this location is desert dry and cold BsKw (wi) according to Köppen as modified by García [27]. The average annual temperature is 17.8 °C, and the average annual precipitation is 341 mm. The driest month is March (average of 6 mm), and most of precipitation falls in June (average 67 mm).

### 2.2. Experimental Design

Multiparous mixed-breed goats from the INIFAP confined flock typical of the extensive farming systems of northern Mexico were used. Thirty non-pregnant three-year-old, non-lactating, and healthy experimental goats [34.4 ± 0.3 kg] were randomly allotted, based on a similar live weight into three groups with (*n* = 10 per group), 0, 30, and 60% *T. recurvata* in the diet (dry matter basis; Table 1). Initial live weight, metabolic profile, and complete blood counts (CBC) of the experimental goats are presented in Table 2.

The experiment was conducted from January to April. The experimental goats were separated and kept away from bucks and were managed under natural climate and photoperiod (22° N). The experimental goats were dewormed before the experiment (−15 d) with 1 g ivermectin (Baymec^®^; Bayer, Morelia, México). Additionally, all experimental goats received I.M. 0.005 g of vitamin B12 (Catosal^®^; Bayer, Morelia, Mexico), 500,000 IU of vitamin A, 75,000 IU of vitamin D_3_, and 50 mg of vitamin E (Vigantol^®^; Bayer, Morelia, Mexico).

### 2.3. Tillandsia recurvata

*T. recurvata* is abundant in the study area [22], and tassels of this epiphyte were manually harvested from *Prosopis* spp. trees heavily infested with this epiphyte every day (~150 kg). Tassels were sun-dried for two days and subsequently transferred to a digital oven (FELISA^®^, Jalisco, México) at 65 °C for 24 h. Dry samples were ground in a mill through a 3 mm screen (NOGUEIRA^®^, São Paulo, Brasil) before being incorporated into the diet. A fresh sub-sample of *T. recurvata* was taken to a laboratory to determine the nutrient content of this plant. The nutritional composition of experimental diets and some mycotoxins content of *T. recurvata* was assessed by AGROLAB Mexico S.A. de C.V. (Table 1). Samples were analyzed according to our previous report [23]. In brief, a computer-based NIRS method was used to analyze the samples of *T. recurvata* [NIRS 5000, FOSS^®^, MD, USA].

### 2.4. Experimental Diets

Experimental diets for the maintenance of non-pregnant, non-lactating goats with minimal physical activity were formulated. Diets included ground sorghum grain, sorghum stubble, soybean meal, and *T. recurvata* (Table 1) and were intended to provide around 7% crude protein and 2 megacalories [Mcal]/kg DM of metabolizable energy [ME] [28]. *T. recurvata* and sorghum stubble were changed based on the diet treatment and modified the dry matter [DM] and energy content. Control goats (T0; *n* = 10) did not receive *T. recurvata*, whereas goats in T30 and T60 groups were offered diets with either 30 or 60% *T. recurvata* (Table 1).

*T. recurvata* was gradually added to the basal diet and goats had 14 days for adaptation to diets and facilities (d-14). Experimental diets were offered for 75 d (900 h) and adjusted every 14 d as per live weight. For this purpose, goats were placed in individual pens (1.5 m × 1.5 m × 1.0 m) for no more than 1 h and a half, until all feed was consumed. Goats spent the rest of the day in a standard pen where clean water was provided without any additional feed or mineral supplement. Feed refusals were quantified on an individual pen basis, however after the two first days of the adaptation period, all feed offered was consumed. Therefore, for the control treatment, the average feed intake was 0.92 kg DM and 1.38 Mcal of ME per animal/day; for T30, the intake was 0.82 kg of DM and 1.05 Mcal of ME per animal/day, and for T60, feed intake was 0.71 kg of DM and 0.77 Mcal of ME per animal/day. The live weight of goats was recorded before feeding on several occasions across the experiment. Goats were weighed with an Ohaus Defender^®^ digital scale with a 150 kg capacity and minimal graduation of 50 g. The data obtained were used to estimate goats’ weight changes.

### 2.5. Blood Sampling and Metabolic Profile

A blood sample (5 mL) from each goat was obtained on days −15, 28, and 56 of the experimental period. Blood was collected from goats via jugular venipuncture into sterile evacuated tubes with sodium heparin (Vacutainer; Franklin Lakes, NJ, USA) just before the morning feeding. Blood samples were placed in the fridge (4 °C) and taken to the lab on ice for analysis five hours later. Plasma was harvested by centrifugation at 1850× *g* for 20 min at 4 °C.

Blood plasma was used to determine all goats’ metabolic profile (total proteins, glucose, triglycerides, phosphorus, and calcium). Plasma glucose and triglycerides were determined by the Trinder method [29,30]; total proteins were determined by the Goldberg method [31]; calcium was determined by the colorimetric method [31], and phosphorus was determined by the UV method [30].

Blood for complete blood counts (CBC) was collected from each goat on −15 d and 56 d by venipuncture in 5 mL evacuated collection tubes containing potassium ethylene diamine tetraacetic acid as an anticoagulant. These samples were used for manual CBC. CBC was quantified with hematocrit by the Wintrobe method; hemoglobin by the oxyhemoglobin method; red blood cells, white blood cells, mean corpuscular volume (MCV), mean corpuscular hemoglobin (MCH) and mean corpuscular hemoglobin concentration (MCHC) were determined following the methods of Jones and Allison [32].

### 2.6. Data Analyses

Statistical analyses were performed using JMP Star Statistics version 4.0.3 Academic [33]. The experiment was organized in a completely randomized design. For all data measurements, individual goats were used as experimental units. Live weight data were analyzed using general linear model procedures. Dietary treatment was considered as fixed effect, and time (sampling dates 1 to 6) as repeated measure over time. The models included the fixed effects of dietary treatment, time (sampling dates as six repeated measures), and the treatment × time interaction.

Metabolic profile data (protein, glucose, triglycerides, calcium, and phosphorus concentration) were analyzed using a fixed model. Dietary treatment was considered as fixed effect, and time (28 and 56 days of the experimental period) as repeated measure over time. Hematic biometry data (hematocrit, hemoglobin, red blood cells, white blood cells, MCV, MCH, and MCHC) were analyzed using a completely random design. The models included the fixed effects of dietary treatment. A Tukey test was used to test differences among sample means for significance when statistical differences were detected (*p* < 0.05).

## 3. Results

### 3.1. Mycotoxins Analyses of Tillandsia recurvata

Some mycotoxins content of *T. recurvata* is presented in Table 3.

### 3.2. Daily Live Weight Change

**Figure 1:**
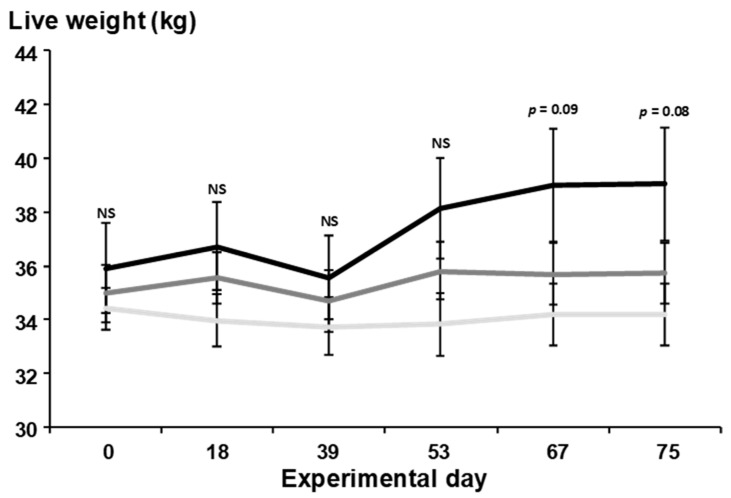
Effects of substituting sorghum stubble for *T. recurvata* on goat live weight. Mean live weight from 0 d to d 75 of the experimental period from multiparous mixed-breed goats that received 0% (T0; solid black line), 30% (T30; solid dark grey line), or 60% *T. recurvata* (T60; solid light grey line) in the diet. NS = non-significant. Values are means ± SEM.

The mean live weight of goats at d 0 was 35.9 ± 1.7 kg for T0, 35.0 ± 1.1 kg for T30, and 34.4 ± 0.7 kg for T60. Final body weight tended (*p* = 0.08) to be heavier for the T0 fed goats (39.1 ± 2.1 kg) compared with T30 (35.7 ± 1.1 kg) and T60 (34.2 ± 1.1 kg; Figure 1). Average liveweight change was greater (42 ± 12 g day^−1^; *p* < 0.01) for T0, compared with T30 and T60 fed goats (10 ± 10 g day^−1^ and −3.0 ± 8 g day^−1^, respectively). Across the experiment, the effect of the time on live weight was significant (*p* < 0.01), increasing from 35.1 ± 0.7 kg at day 0 to 36.3 ± 0.9 kg at the end, although the effect of the treatment was not significant (*p* > 0.05). The interaction between dietary treatment and time was significant (*p* < 0.05). Live weight increased in T30, whereas in T60, it was reduced by 1% (Figure 1).

### 3.3. Metabolic Profile

The metabolic profile of goats offered different levels of *T. recurvate,* and this is presented in Table 4. In all treatments, as the trial progressed, plasma glucose concentration decreased 16% from d 28 to d 56 (57.8 mg dL^−1^ to 48.6 mg dL^−1^; *p* < 0.001); whereas plasmaphosphorus concentration decreased 11% from d 28 to d 56 (5.6 mg dL^−1^ to 5.0 mg dL^−1^; *p* < 0.001). Plasma triglycerides concentration increased 13% from d 28 to d 56 (62.6 mg dL^−1^ to 70.7 mg dL^−1^; *p* < 0.001); whereas calcium concentration increased 9.5% from d 28 to d 56 (7.6 mg dL^−1^ to 8.4 mg dL^−1^; *p* < 0.01). Collection time did not affect the plasma total protein (*p* > 0.05; Table 4). Plasma total protein, glucose, triglycerides, phosphorus, and calcium concentration did not differ among treatments (*p* > 0.05; Table 4). The interaction between treatment and period was not significant for all the variables tested (*p* > 0.05; Table 4).

### 3.4. Complete Blood Count

The mean of the blood components of the goats within treatment is presented in Table 4. The MCV level was higher (*p* < 0.001) in T0 goats than T30 and T60 animals (Table 5). Similarly, the MCH was higher (*p* < 0.05) in T0 goats than in T60 animals (Table 5). White blood cells presented higher (*p* < 0.05) counts in T30 goats than in T0 and T60 goats, and hematocrit tended (*p* < 0.06) to differ among treatments with the lowest value for T60 goats (Table 5). Red blood cells presented higher (*p* < 0.01) counts in T60 goats compared to T30 animals, with T0 goats showing as intermediate (Table 5); whereas the rest of the variables tested did not differ among dietary treatments (*p* > 0.05; Table 5).

## 4. Discussion

The results demonstrated two interesting facts: (1) the replacement of sorghum stubble with 30% *T. recurvata* resulted in a slight weight gain, however, total replacement of sorghum stubble with *T. recurvata* resulted in a 1% reduction in the live weight of goats without negative effects on metabolic profile; and (2) mycotoxins of *T. recurvata* were within safe levels for livestock, and thus, they did not represent a pathogenic concern for goats. The ingestion of feeds naturally contaminated with mycotoxins has been shown to decrease livestock performance, leading to decreased feed intake and weight gain and adverse metabolic, hematological, and neurochemical changes [34,35]. However, in the present study, no effects of various mycotoxins on serum chemistry or hematology were observed when goats were fed *T. recurvata* naturally contaminated with mycotoxins. Certainly, the T30 diet helped goats maintain live weight and even gain weight by the end of the experiment. Therefore, the inclusion of up to 30% of *T. recurvata* in the diet can be used as alternative forage in pen-fed goats in the semi-arid environment of northern Mexico.

Towards the end of the experiment, live weight of goats changed positively in T0 and T30 goats; however, goats in the T60 group ended the trial with 1% live weight loss. T0 goats gained more weight than T30 or T60. This can be explained by the differences in ME, and DM content of the experimental diets, as *T. recurvata* provides less ME [0.58 Mcal/kg vs. 1.95 Mcal/kg DM basis] and DM [53% vs. 88%] than sorghum stubble [23,28]. Interestingly, T0 and T30 goats gained weight towards the end of the trial despite being offered maintenance diets. We previously observed the same response with experimental goats indicating a possible adjustment in their metabolism and positive energy balance [11]. Importantly, *T. recurvata* in the diet was ground to facilitate its consumption, although, under extensive conditions, goats ingest the whole plant of *T. unsenoides* [6].

The metabolic profile of T30 and T60 goats indicated that they were in a positive energy balance. The observed plasma glucose and triglycerides concentrations were within and above the reference values for goats [36,37,38]. However, towards the end of the experiment, plasma glucose concentration of goats from all dietary treatments decreased, whereas live weight increased. Furthermore, plasma triglyceride levels increased at the end of the trial. The decrease in plasma glucose levels and increase in plasma triglycerides were probably due to an adjustment to a new set point in maintaining glucose homeostasis as this effect occurred in all treatments. Blood glucose and triglyceride concentrations are good indicators of the energy balance of animals [39]. These results highlight the ability of goats to adapt both their feed intake and metabolism to the feed offered regardless of their nutrient requirements [6,10,12,40]. Nevertheless, the inclusion of 30% *T. recurvata* in the diet resulted in a modest live weight gain, whereas 60% *T. recurvata* in diets slightly reduced live weight without variation in their plasma metabolites indicative of body energy reserves in these mixed-breed goats.

The plasma total protein levels of goats from this experiment were below the range reported for Criollo × Canaria, and Granadina breeds in Venezuela and México, respectively [41,42]. In the current study, as the experiment progressed and although no statistical difference existed, plasma total protein decreased in all the dietary treatments, and moreover, the level was lower in the goats that consumed *T. recurvata* than the control goats, despite the fact that the crude protein content of *T. recurvata* is similar to sorghum stubble [23,28]. Indeed, plasma protein concentration can be manipulated by nutritional diet [43], and the diet offered to goats in the present experiment was not similar to those studied by Matheus and Figueiredo [41] or Mellado et al. [42]. Extending the observations of Harmeyer and Martens [43], the difference observed in plasma total protein concentration in the present experiment could be due to the limited crude protein of diets formulated only for maintenance. Nevertheless, if goat producers decide to include *T. recurvata* in the diet, an additional source of protein will be necessary to reach adequate weight gains and increase reproductive efficiency under extensive conditions.

Almost all blood variables were within the normal range, however, the number of red blood cells in T0 and T30 goats was below the normal range [44]. Yet, towards the end of the experiment, red blood cell counts in the T60 goats were above the normal range. We previously demonstrated that the mineral content of *T. recurvata* was within the normal range for good forages; however, the levels of iron were higher than in other forages [23,45]. Therefore, we hypothesized that the inclusion of 60% of *T. recurvata* in a diet would help to increase blood iron levels and eventually prevent anemia [46,47]. Anemia is caused by a reduction in red blood cells, which in turn can be caused by reduced iron intake in the absence of specific diseases [48,49]. Further research is warranted to determine if *T. recurvata* increases blood iron levels in goats. However, if our results are correct, these observations are very relevant to the arid and semiarid areas of the world as the current results suggest that in this semi-arid area, with a high abundance of *Tillandsia* spp., the ingestion of this epiphyte markedly increased red blood cells, which could be linked to the high iron provided by this plant. Limited nutrients available is a recurrent scenario observed in marginal small ruminant production systems [1,6,12,50]

Moreover, we observed an increase in the mean corpuscular volume (MCV) and mean corpuscular hemoglobin (MCH) in T0 goats. These values were higher than those reported by Antunović et al. [51] in female sheep at different physiological states. We do not have an explanation for this response as high MCV levels above some critical value in animals suggests the induction of regenerative anemia. Still, no anemia was detected in these goats regardless of the percentage of *T. recurvata* ingested. In addition, the hematocrit values in the goats from the current experiment were adequate and it has been indicated that normal hematocrit values indicate sufficient red blood cell levels [52]. Finally, it is worth noting that a higher MCV value is not always associated with a pathologic condition [53].

In northern Mexico′s arid and semi-arid zones, most sheep and goats kept on rangeland do not receive mineral supplementation throughout their lives, even during lactation; therefore, a mineral deficiency could be alleviated by the inclusion of *T. recurvata* in the diet. Goats consume *Tillandsia* spp. during the dry season [6]; therefore, the inclusion of *T. recurvata* in the diet would be more beneficial during the dry season when most forages are dry and scarce. From an economic point of view, the use of *T. recurvata* in goat nutrition is cheap, and from an ecological point of view, harvesting *T. recurvata* to feed goats will be a natural way to control the damage caused by this aggressive epiphyte to mesquite [*Prosopis glandulosa*] or any other host trees [21]. Further research is necessary to elucidate this view.

The protein content of the experimental diets satisfied the maintenance requirements for goats with low physical activity [28]. The study aimed to find a way to improve the nutritional status of goats kept on semi-arid rangeland or similar ecosystems in other parts of the world. On a global scale, goat production is carried out mainly in extensive grazing systems, where the forage supply generally does not satisfy the nutritional needs of the animals [6]. We, therefore, decided to use animals in the field to mimic the real-world situation. The low protein content of the experimental diets is consistent with the absence of marginal weight gain obtained during the study.

It is possible that the positive effect of the experimental diets on the plasma metabolites profile and blood components of goats could have been due to the inclusion of sorghum grain and soybean meal, as both ingredients provide substantial metabolizable energy and crude protein in the diet. Indeed, *T. recurvata* contains adequate levels of metabolizable energy, crude protein, and dry matter for non-pregnant, non-lactating small ruminants on a maintenance diet with low physical activity [23,28]; yet, dry matter intake of T60 goats was 30% lower than T0, which suggests that secondary metabolites of *T. recurvata* hamper feed intake, which might explain the lack of weight gain in T60 goats.

The potentially toxic components (mycotoxins) of *T. recurvata* were below the limit of toxicity [54]. Extending our results, several authors have indicated that animal performance is not affected when consuming feed with low to moderate secondary compounds [6,55,56]. Yet, in plants, Valencia-Díaz et al. [57] indicated that *T. recurvata* has phytotoxic properties, which inhibit seed germination of its nearest neighbor. In the present study, 60% of *T. recurvata* in the diet did not affect the blood metabolites and components of goats, perhaps by the trial duration. However, there are more plant secondary compounds identified in plants, thus, the estimation of a determined number of them does not preclude the presence of others with a variety of effects over small ruminants [58]. Nevertheless, to clarify the toxic effect of the consumption of this epiphyte on goats, further research would be necessary to determine their secondary compounds and to assess some liver enzymes in the blood indicative of liver damage for detection of toxicity.

## 5. Conclusions

Replacing sorghum stubble with different levels of *T. recurvata* in the diet of non-lactating adult mixed-breed goats adversely affected weight change, although these changes were not high. The inclusion of *T. recurvata* in the diet did not modify the goats’ metabolic status and increased the counts of red and white blood cells of the goats, although no signs of toxicity were detected. Therefore, this epiphyte can be a safe feedstuff to use in goats on rangeland as a feed supplement to improve the income of goat producers without significantly increasing the demands on feed, and, at the same time, it would reduce the damage caused by *T. recurvata* to host trees in semi-arid rangelands.

## Figures and Tables

**Table 1:** Ingredients and chemical composition of experimental diets (DM basis) containing different levels of *Tillandsia recurvata*.

Ingredient Composition (% in Diet)	Treatment			DM (%)	CP (%)	ME (Mcal)
	T0	T30	T60			
Ground sorghum grain	32	32	32	88	7.7	3.07
Soybean meal	8	8	8	89	30.4	4.99
*Tillandsia recurvata*	0	30	60	53	6.1	0.58
Sorghum stubble	60	30	0	88	5.2	1.95
Nutrient composition experimental diet						
Crude protein, %				6.58	6.91	7.25
Metabolizable energy (Mcal kg^−1^ DM)				1.49	1.29	1.09

**Table 2:** Initial (−15 d) live weight, metabolic profile, and complete blood count of mixed-breed goats receiving 0% (T0), 30% (T30), or 60% (T60) of *T. recurvata* in the diet.

Variable	Dietary Treatment		
	T0	T30	T60
Live weight (kg)	34.4 ± 0.6	34.2 ± 0.5	34.5 ± 0.5
	Metabolic Profile		
Total proteins (g dL^−1^)	6.01 ± 0.42	6.03 ± 0.01	6.08 ± 0.57
Glucose (mg dL^−1^)	62.5 ± 8.2	59.2 ± 3.5	56.3 ± 2.2
Triglycerides (mg dL^−1^)	57.7 ± 11.7	53.9 ± 5.9	55.9 ± 10.6
Calcium (mg dL^−1^)	8.4 ± 0.1	8.4 ± 1.5	8 ± 0.6
Phosphorus (mg dL^−1^)	5.8 ± 0.7	5.9 ± 0.6	6.1 ± 1.3
	Complete Blood Count		
Hematocrit (%)	33.3 ± 4.9	38.7 ± 6.0	33.2 ± 4.5
Hemoglobin (g/dL)	10.1 ± 3.0	10.0 ± 2.6	10.4 ± 1.7
Red blood cells (10^6^/mL)	5.1 ± 0.9	5.2 ± 1	4.7 ± 1.1
White blood cells (10^3^/mL)	11.6 ± 2.6	9.7 ± 4.7	10.2 ± 3.9
MCV (fl)	20.2 ± 2.8	19.2 ± 2.9	19.5 ± 2.8
MCH (pg/cell)	21.1 ± 9.3	19.8 ± 6.7	23.2 ± 6.4
MCHC (%)	30.4 ± 8.7	26.7 ± 8.9	31.5 ± 7.4

MCV: Mean Corpuscular Volume; MCH: Mean Corpuscular Hemoglobin; MCHC: Mean Corpuscular Hemoglobin Concentration. Values are means ± SEM.

**Table 3:** Some mycotoxins of powdered *T. recurvata*.

Secondary Metabolite	As Sampled Basis	Dry Matter Basis
Dry Matter (%)	46.3	
Aflatoxins, mg/g (10^−9^)	4.5	9.7
Zearalenone, mg/g (10^−9^)	45.4	98.2
Deoxynivalenol, mg/kg	1.36	2.93
Trichothecene, mg/kg (10^−9^)	65.4	141.3

**Table 4:** The effect of the dietary replacement of sorghum stubble for *T. recurvata* on the plasma total proteins, glucose, triglycerides, calcium, and phosphorus concentration of mixed-breed goats within dietary treatment (TRT) on d 28 and d 56 of the experimental period and receiving 0% (T0), 30% (T30) or 60% (T60) of *T. recurvata* in the diet. Values are means ± SEM. Data for treatment analysis combined both periods. Data for period analysis combined all treatments.

Variable	TRT	Experimental Day		Significance		
		28	56	TRT	Time (T)	TRT × T
Total proteins (g dL^−1^)	T0	6.19 ± 0.13	6.11 ± 0.12	NS	NS	NS
	T30	5.74 ± 0.11	5.66 ± 0.11			
	T60	5.86 ± 0.12	5.6 ± 0.2			
Glucose (mg dL^−1^)	T0	60.3 ± 4.04	49.9 ± 2.64	NS	***	NS
	T30	55.7 ± 3.42	51.9 ± 3.02			
	T60	57.5 ± 3.18	44.2 ± 2.42			
Triglycerides (mg dL^−1^)	T0	61.9 ± 1.6	71.8 ± 2.1	NS	***	NS
	T30	63.1 ± 3.5	69.2 ± 1.65			
	T60	63 ± 2.6	71.2 ± 2.05			
Calcium (mg dL^−1^)	T0	7.49 ± 0.2	8.64 ± 0.34	NS	**	NS
	T30	7.91 ± 0.3	8.58 ± 0.23			
	T60	7.56 ± 0.2	7.92 ± 0.34			
Phosphorus (mg dL^−1^)	T0	5.63 ± 0.25	5.65 ± 0.23	NS	***	NS
	T30	5.64 ± 0.26	4.8 ± 0.13			
	T60	5.6 ± 0.37	4.62 ± 0.17			

NS: Non-significant; ** *p* < 0.01; *** *p* < 0.001. Values are means ± SEM. Data for treatment analysis combined both periods. Data for period analysis combined all treatments.

**Table 5:** Blood components of mixed-breed goats fed different levels of *T. recurvata* on day 56 of the trial. Values are means ± SEM.

Variable	Day of Feeding Trial			Significance
	56			
	T0	T30	T60	
Hematocrit (%)	32.2 ± 1.53	35.3 ± 1.52	29.9 ± 1.64	0.06
Hemoglobin (g/dL)	11.3 ± 0.89	10.3 ± 0.58	9.0 ± 0.59	NS
Red blood cells (10^6^/mL)	6.64 ± 0.75 ^ab^	5.89 ± 0.37 ^b^	9.44 ± 1.25 ^a^	**
White blood cells (10^3^/mL)	9.27 ± 0.79 ^b^	12.0 ± 0.71 ^a^	9.34 ± 1.08 ^b^	*
MCV (fl)	45.3 ± 4.98 ^a^	21.0 ± 0.71 ^b^	23.4 ± 0.55 ^b^	***
MCH (pg/cell)	19.6 ± 3.14 ^a^	18.7 ± 1.61 ^a^	10.7 ± 1.34 ^b^	*
MCHC (%)	34.4 ± 2.49	30.1 ± 2.01	30.5 ± 1.89	NS

Abbreviations: MCV: Mean corpuscular volume; MCH: Mean corpuscular hemoglobin; MCHC: Mean corpuscular hemoglobin concentration. NS: Non-significant; * *p* < 0.05; ** *p* < 0.01; *** *p* < 0.001. ^a,b^ Values within the same variable and period with a different superscript letter differ (*p* < 0.05).

## Data Availability

The data presented in the manuscript is part of the Ph.D. thesis entitled “Evaluación de la calidad nutrimental de *Tillandsia recurvata* para la suplementación de cabras en época de estiaje”, in which the first author of the manuscript, Héctor G. Gámez-Vázquez, is the author of the thesis. The thesis was submitted to the Universidad Autonoma de Zacatecas (Autonomous University of Zacatecas), México.
